# SOCS3 Acts as an Onco-immunological Biomarker With Value in Assessing the Tumor Microenvironment, Pathological Staging, Histological Subtypes, Therapeutic Effect, and Prognoses of Several Types of Cancer

**DOI:** 10.3389/fonc.2022.881801

**Published:** 2022-05-06

**Authors:** Lirui Dai, Yiran Tao, Zimin Shi, Wulong Liang, Weihua Hu, Zhe Xing, Shaolong Zhou, Xuyang Guo, Xudong Fu, Xinjun Wang

**Affiliations:** ^1^ Institute of Neuroscience, Zhengzhou University, Zhengzhou, China; ^2^ Department of Neurosurgery, The Fifth Affiliated Hospital of Zhengzhou University, Zhengzhou University, Zhengzhou, China; ^3^ Henan International Joint Laboratory of Glioma Metabolism and Microenvironment Research, Zhengzhou, China

**Keywords:** *SOCS3*, immuno-oncology, gene expression profiling, immune-cell infiltration, genetic and epigenetic alterations, immunotherapy, chemotherapy, biomarker

## Abstract

The suppressor of cytokine signaling (*SOCS*) family contains eight members, including *SOCS1–7* and *CIS*, and *SOCS3* has been shown to inhibit cytokine signal transduction in various signaling pathways. Although several studies have currently shown the correlations between *SOCS3* and several types of cancer, no pan-cancer analysis is available to date. We used various computational tools to explore the expression and pathogenic roles of *SOCS3* in several types of cancer, assessing its potential role in the pathogenesis of cancer, in tumor immune infiltration, tumor progression, immune evasion, therapeutic response, and prognostic. The results showed that *SOCS3* was downregulated in most The Cancer Genome Atlas (TCGA) cancer datasets but was highly expressed in brain tumors, breast cancer, esophageal cancer, colorectal cancer, and lymphoma. High *SOCS3* expression in glioblastoma multiforme (GBM) and brain lower-grade glioma (LGG) were verified through immunohistochemical experiments. GEPIA and Kaplan–Meier Plotter were used, and this bioinformatics analysis showed that high *SOCS3* expression was associated with a poor prognosis in the majority of cancers, including LGG and GBM. Our analysis also indicated that *SOCS3* may be involved in tumor immune evasion *via* immune cell infiltration or T-cell exclusion across different types of cancer. In addition, *SOCS3* methylation was negatively correlated with *mRNA* expression levels, worse prognoses, and dysfunctional T-cell phenotypes in various types of cancer. Next, different analytical methods were used to select genes related to *SOCS3* gene alterations and carcinogenic characteristics, such as *STAT3*, *SNAI1*, *NFKBIA*, *BCL10*, *TK1*, *PGS1*, *BIRC5*, *TMC8*, and *AFMID*, and several biological functions were identified between them. We found that *SOCS3* was involved in cancer development primarily through the *JAK/STAT* signaling pathway and cytokine receptor activity. Furthermore, *SOCS3* expression levels were associated with immunotherapy or chemotherapy for numerous types of cancer. In conclusion, this study showed that *SOCS3* is an immune-oncogenic molecule that may possess value as a biomarker for diagnosis, treatment, and prognosis of several types of cancer in the future.

## Introduction

Cancer is currently the primary cause of death worldwide, and its morbidity and mortality rates have been increasing on an annual basis. The latest research released by the International Agency for Research on Cancer indicated that lung cancer remains the most common cause of cancer-associated death, followed by colorectal cancer and prostate cancer (1). Therefore, there is an urgent need for potential diagnostic markers and therapeutic methods to control tumor progression.

Tumorigenesis is a complex process involving genetic alterations and epigenetic modifications (2). Genetic and epigenetic alterations can alter the progression of cancers and promote tumor progression (3). The tumor microenvironment (TME) is widely associated with the occurrence of cancers. Immune cells, fibroblasts, adipocytes, and the tumor vascular systems all contribute to the tumor microenvironment (4). Tumor-associated immune cells can be roughly segmented into two types: tumor-antagonistic immune cells and tumor-promoting immune cells. The two types of cells perform different functions at different stages of tumor formation (5). It is worth mentioning that tumor-promoting immune cells can affect immune homeostasis and immune tolerance (5), promote tumor cell migration to endothelial cells, increase metastasis (6), promote immune avoidance, increase tumor blood vessel formation, and promote the acquisition of treatment resistance, and these processes involve manipulation of regulatory T cells (Tregs), myeloid-derived suppressor cells (MDSC), cancer-associated fibroblasts (CAF), and M2-polarized macrophages (7).

Different types of immune-related cells serve differing roles in various types of cancer; therefore, immune escape of tumors has become a challenging problem in disease treatment (5). Through bioinformatics analysis, we concentrated on the immune microenvironment of tumors, investigating multiple tumor immunosuppression mechanisms and therapeutic methods, and provided evidence for cancer diagnosis, therapeutic responses, and prognosis (8).

**Figure 1:**
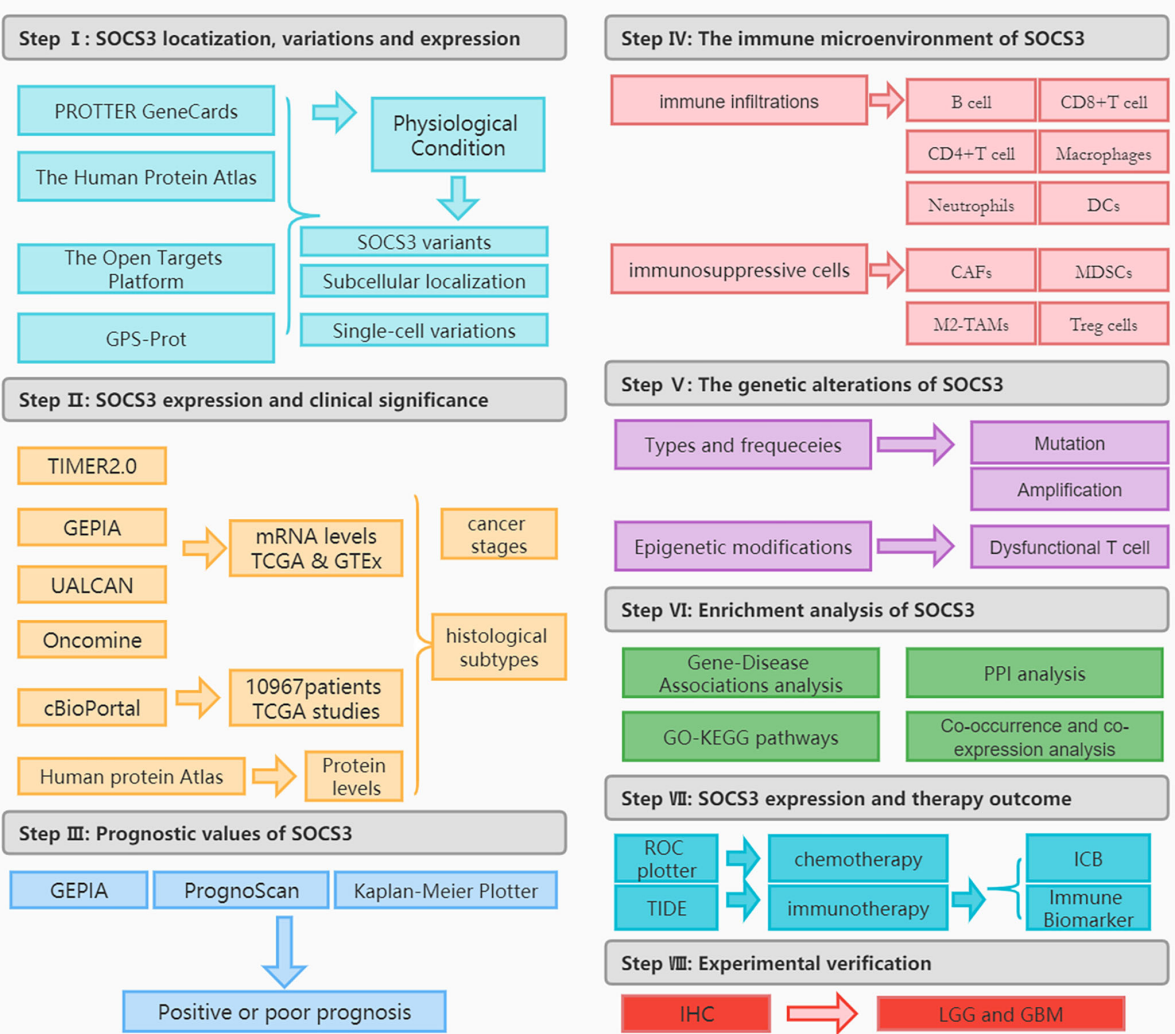
The flowchart of this study.

The *SOCS* family consists of eight members, and *SOCS3* can affect the occurrence of tumors by interacting with a variety of immune molecules and several signaling pathways (9, 10). We previously summarized the role of *SOCS3* in several types of tumors (10), and numerous experiments have also confirmed the association between *SOCS3* and different types of cancer, including breast cancer (11), colorectal cancer (12), and glioblastoma (13). However, the pathogenesis of *SOCS3* in various types of cancer and whether there is a common molecular mechanism between these different types of cancer in regulating the pathogenic effects and treatment responses remain to be further studied. Therefore, we explored the potential molecular mechanisms of *SOCS3* in the diagnosis, treatment, and prognosis of a range of cancers (**Figure 1**).

## Materials and Methods

### Gene Expression Analysis

Oncomine (https://www.oncomine.org/) (access date: November 1, 2021) was employed to investigate the *mRNA* expression levels of *SOCS3* in several types of cancer (14). The threshold values were set in line with the following parameters: analysis of components, cancer *vs*. normal tissue and cancer *vs*. cancer tissue; *p-*value, 0.01; gene ranking of top 10%; and fold change, all. A t-test was used to calculate the *p*-value. Furthermore, the TIMER2.0 (http://timer.cistrome.org/) (access date: November 2, 2021) database (15), gene expression profiling interactive analysis (GEPIA2) database (http://gepia2.cancer-pku.cn/) (access date: November 3, 2021) (16), and UALCAN (http://ualcan.path.uab.edu/) (access date: November 3, 2021) (17) were employed to systematically compare the expression levels of *SOCS3* between cancers and the corresponding normal tissues. We used the GEPIA2 and UALCAN databases to assess the *SOCS3* expression in different tumor pathological stages across TCGA cancer types. *p*<0.05 was considered to indicate a statistically significant difference.

### Survival Prognosis Analysis

GEPIA2 and Kaplan–Meier plotter (http://www.kmplot.com/analysis/) (access date: November 4, 2021) (18) were used to investigate the overall survival (OS) and disease-free survival (DFS) of *SOCS3* based on TCGA data. Cutoff-low (50%) and cutoff-high (50%) values were used as the different expression thresholds. Patient samples were segmented into high and low *SOCS3* expression groups and analyzed using the risk ratio (95% confidence interval) and log rank test *p*-values.

### Tumor Immune Cell Infiltration (TIIC) Analysis

The association between the *SOCS3* gene and tumor immune cell infiltrations (TIICs) (including B cells, CD8+ T cells, CD4+ T cells, DC cells, neutrophils, and macrophages) in different types of tumors were investigated using the TIMER2.0 database. Spearman-based correlation analysis was corrected based on tumor purity (19). The correlation of *SOCS3* expression with several immunosuppressive cells, which can promote T-cell exclusion, were analyzed, including the M2 subtype of tumor-associated macrophages (M2-TAMs), cancer-associated fibroblasts (CAFs), myeloid-derived suppressor cells (MDSCs), and regulatory T (Treg) cells in 40 different cancers based on data obtained from TCGA. The Spearman’s rho value was corrected using tumor purity, and p<0.05 was considered to indicate a statistically significant difference. Furthermore, we applied Tumor Immune Dysfunction and Exclusion (TIDE) (http://tide.dfci.harvard.edu/) (access date: November 4, 2021) to assess whether the epigenetic alterations of *SOCS3* could impact the dysfunctional T-cell phenotypes (20, 21). We visualized the levels of immune cell infiltration using heatmaps *via* 35 cancer types.

### Gene Alteration Co-Occurrence and Gene Co-Expression Analysis

We employed the cBioPortal (http://www.cbioportal.org/) (access date: November 5, 2021) database to evaluate co-occurrence modes of gene mutation between *SOCS3* and other genes based on data from 10,967 patients from TCGA Pan-Cancer Atlas Studies (22). Co-occurrence modes of gene mutations were directed as follows: log ratio >5, *p-*value < 1 ×10^−10^. The Oncomine database was used to assess related genes that were associated with *SOCS3* expression in different types of tumors.

### Epigenetic Methylation Analysis

The methylation section of the UALCAN database was used to investigate differences in *SOCS3* methylation status between different types of tumors and normal tissues based on TCGA cancer types (23). β-Values represent the promoter methylation levels, ranging from 0 to 1, which corresponded to unmethylated and fully methylated, respectively. Hypomethylation was considered as β-values in the range of 0.25–0.3, whereas hypermethylation was considered as β-values in the range of 0.5–0.75 (24).

### *SOCS3*-Related Gene Enrichment and PPI Network Analysis

The GPS-Prot (http://gpsprot.org/) (access date: November 5, 2021) database was applied for protein–protein interaction (PPI) analysis of *SOCS3* (25), while the STRING (https://string-db.org/) (access date: November 6, 2021) website was used to determine the proteins interacting with *SOCS3* (26). These databases constructed PPI networks based on the co-expression analysis, colocalization, genetic interaction, and common pathways. We employed the GEPIA2 database to gain the top 100 target genes related to *SOCS3* and conducted Pearson correlation analysis, respectively. In addition, we used the TIMER2.0 database to obtain the heatmap data of the six top-most related genes.

We used Jvenn, an interactive Venn graph viewer, to compare the genes that interacted with *SOCS3* (27). Moreover, PPI and enrichment analyses were performed using the *SOCS3* co-expressed and co-mutated genes. We used Database for Annotation, Visualization, and Integrated Discovery (DAVID) (https://david.ncifcrf.gov/) (access date: November 7, 2021) to perform Gene Ontology (GO) and Kyoto Encyclopedia of Genes and Genomes (KEGG) enrichment analysis. The enrichment *p*-value <0.05 was considered to indicate a statistically significant difference (28).

### Correlation Analysis of Gene Expression With Therapeutic Effect, Genetic Priority, and Standardized Biomarkers

We used the ROC plotter (http://rocplot.org/) (access date: November 9, 2021) database to analyze whether *SOCS3 mRNA* expression was relevant to therapeutic responses in patients with breast cancer, ovarian cancer, glioblastoma (GBM), and colorectal cancer (29). In addition, we used the TIDE website and queried the regulator prioritization of *SOCS3* from four aspects, which specifically included the response to T dysfunction, immune checkpoint blockade therapy (ICB), CRISPR screens, and immune-suppressive cell types. It is worth mentioning that the Z-score in the Cox-PH regression was employed to assess whether differences in *SOCS3* expression had influences on ICB-treated patients. Furthermore, we entered the biomarker evaluation section of the TIDE to compare the therapeutic responses of SOCS3 expression to different cancer types with nine standardized tumor immune response biomarkers, including *MSI*, *TMB*, *CD274*, *CD8*, *IFNG*, *T.Clonality*, *B.Clonalityand*, *Merck 18*, and *TIDE* (20, 21).

### Immunohistochemistry Staining

We fixed tissues obtained from 30 HGG patients (11 GBM) and 32 LGG patients with 10% formaldehyde, which were subsequently paraffin-embedded and sectioned, and then, tissues were incubated overnight at 4°C with an anti-*SOCS3* antibody (Abcam, cat. no. AB16030, 1:100 dilution). After washing with phosphate-buffered saline (PBS), we added sheep anti-rabbit secondary antibody (1∶200 dilution) labeled with horseradish peroxidase (HRP) and incubated at room temperature for 20 min. Samples were washed again with PBS, and HRP-conjugated streptomycin working solution was added and incubated at room temperature for 15 min. Then, DAB chromogenic solution was used for color development, and hematoxylin staining was observed under a microscope. The areas with a strong immune response in each section were selected, and five non-repeating fields were observed with a low magnification field (200×) and high magnification field (400×), respectively, and the number of *SOCS3*-positive cells were counted.

## Results

### *SOCS3* Localization, Variations, and Expression Under Physiological Conditions

**Figure 2:**
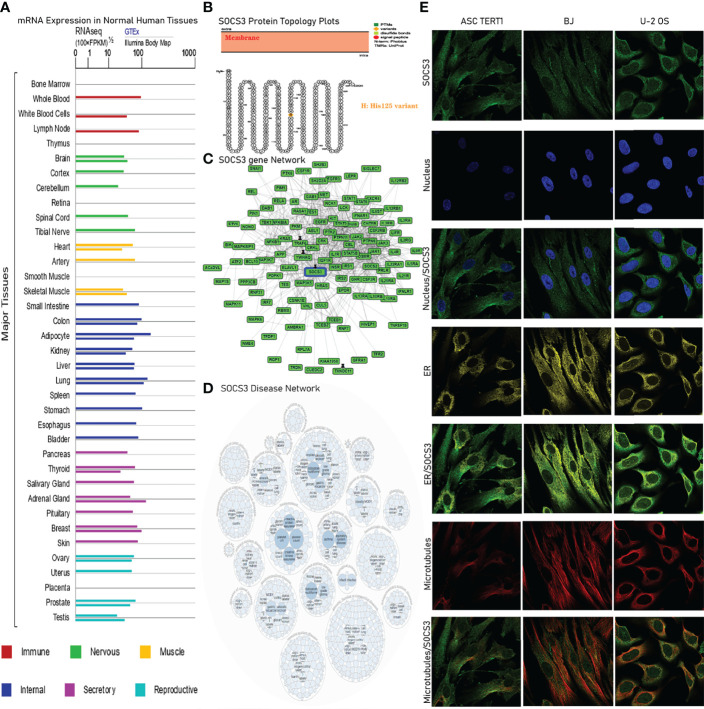
S*OCS3* localization, variations, and expression under physiological conditions. **(A)** *SOCS3 mRNA* expression status in disparate normal human samples across GTEx database. **(B)** *SOCS3* protein topology shows a His125 variant that is located in the membrane. **(C, D)** Network of functional genes and diseases associated with *SOCS3*. **(E)** Immunofluorescence staining of subcellular distribution of *SOCS3* in endoplasmic reticulum (ER), nucleus and microtubule of ASC TERT1, BJ, and U-2 osteosarcoma.

We employed the GeneCards (https://www.genecards.org/) (access date: November 2, 2021) database to explore *SOCS3 mRNA* expression in normal tissues and found that *SOCS3* was highly expressed in the nervous system, reproductive system, immune system, and various visceral tissues (**Figure 2A**) (30, 31). Then, PROTTER (https://wlab.ethz.ch/protter/start/) (access date: November 4, 2021) was used to explore the *SOCS3* protein topology. A natural missense variant of Tis125 was located in the intracellular membrane (**Figure 2B**). Next, the GPS-Prot database was used to display the *SOCS3* gene network and found the genes correlated with *SOCS3* (**Figure 2C**). The Open Targets Platform (https://platform.opentargets.org/) (access date: November 10, 2021) database was applied to analyze the correlations between *SOCS3* and diseases. The results showed that *SOCS3* was associated with benign tumors, cancers, nervous system diseases, respiratory or thoracic diseases, genetic diseases, endocrine system diseases, nutritional or metabolic diseases, immune system diseases, and gastrointestinal diseases (**Figure 2D**). Finally, we used The Human Protein Atlas (https://www.proteinatlas.org/) (access date: November 13, 2021), specifically the Cell Atlas section, to evaluate the distribution of *SOCS3* within the nucleus, microtubules, and endoplasmic reticulum (ER) of ASC TERT1, BJ, and U-2 OS cells *via* immunofluorescence analysis. We found that *SOCS3* was co-localized with microtubules and ER markers in ASC TERT1, BJ, and U-2 OS, which suggested that the subcellular localization of *SOCS3* was largely restricted to microtubules and ER. An overlap between *SOCS* and the nucleus was not observed (**Figure 2E**).

### *SOCS3* Expression Is Associated With Cancer Stage and Histological Subtype

**Figure 3:**
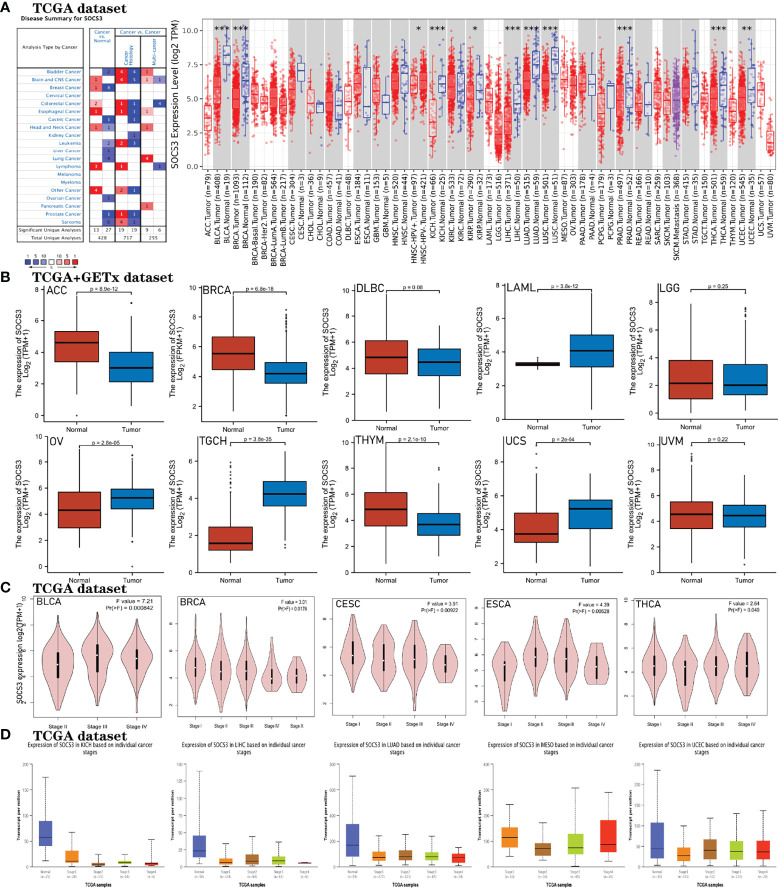
Expression of *SOCS3* in disparate cancers and pathological stages. **(A)** Expression of *SOCS3 mRNA* in disparate kinds of cancers analyzed with Oncomine and TIMER2.0 databases. Oncomine website: fold change = 2, p-value = 0.01. The value in the figure on behalf of the number of datasets that satisfy the threshold. Red and blue indicate the degree of upregulation and downregulation respectively. TIMER2.0: ***p < 0.001, **p < 0.01, *p < 0.05. **(B)** Expression of *SOCS3* mRNA levels in ACC, BRCA, DLBC, LAML, LGG, OV, TGCH, THYM, UCS, and UVM across TCGA cancers, and the corresponding normal samples in the GETx dataset were employed as controls. **(C, D)** GEPIA2 and UALCAN databases were used to analyze the relationship between pathological stages (stage I, II, III, and IV) of various tumors and *SOCS3* expression levels across TCGA data, including BLCA, BRCA, CESC, ESCA, THCA, KICH, LIHC, LUAD, MESO, and UCEC.

The Oncomine website was employed to compare the *mRNA* expression levels of *SOCS3* between various types of cancer and the corresponding healthy samples. The results indicated that the expression of *SOCS3 mRNA* was upregulated in lymphoma, esophageal cancer, and glioma compared with normal tissues, while the expression of *SOCS3 mRNA* was downregulated in bladder cancer, lung cancer, leukemia, and sarcoma compared with the respective normal controls (**Figure 3A**). Next, we used the TIMER2.0 to probe the expression of *SOCS3* across a range of cancers. The results indicated that *SOCS* expression was lower in bladder urothelial carcinoma (BLCA), breast invasive carcinoma (BRCA), kidney chromophobe (KICH), liver hepatocellular carcinoma (LIHC), lung adenocarcinoma (LUAD), lung squamous cell carcinoma (LUSC), prostate adenocarcinoma (PRAD), testicular germ cell tumors (TGCT) (*p*<0.001), uterine corpus endometrial carcinoma (UCEC) (*p*<0.01) and head and neck squamous cell carcinoma (HNSC), and kidney renal papillary cell carcinoma (KIRP) (*p*<0.05) compared with the healthy controls.

**Figure 4:**
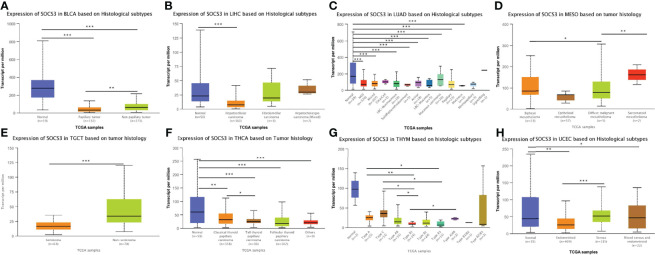
The correlations between *SOCS3* expression levels and histological subtypes in disparate cancers. This figure investigated that the expression levels of *SOCS3* based on histological subtypes in various cancers, such as BLCA, LUAD, THYM, UCEC, LIHC, MESO, TGCT, and THCA, and there was significant statistical significance among multiple groups of data. ***p < 0.001, **p < 0.01, *p < 0.05.

We used normal samples from the GTEx to investigate the expression of *SOCS3* in the corresponding tumors. We found that the expression of *SOCS3* in adrenocortical carcinoma (ACC), acute myeloid leukemia (LAML), ovarian serous cystadenocarcinoma (OV), testicular germ cell tumors (TGCT), thymoma (THYM), and uterine carcinosarcoma (UCS) was statistically significant compared with the corresponding normal tissues (**Figure 3B**). GEPIA2 and UALCAN databases were also employed to evaluate the correlations between *SOCS3* expression and the pathological status of tumors, such as in BLCA, BRCA, Cervical and endocervical cancers (CESC), esophageal carcinoma (ESCA), thyroid carcinoma (THCA), KICH, LIHC, LUAD, mesothelioma (MESO) and UCEC (all *p* < 0.05, **Figures 3C** **, D**). It can be seen that the expression levels of *SOCS3* in the cancers shown in **Figures 3C** **, D** predominantly decreased as the tumor pathological stage increased, consistent with the protein expression levels of *SOCS3* in the corresponding cancers. Furthermore, compared with the expression profiles of cancer histological subtypes, there was a statistically significant difference in *SOCS3* expression among BLCA, LUAD, THYM, UCEC, LIHC, MESO, TGCT, and THCA subtypes, and *SOCS3* expression was higher in all histological subtypes compared with the corresponding normal adjacent tissues in BLCA, LUAD, THYM, and THCA (**Figure 4**; **Table 1**).

**Table 1:** Expression of *SOCS3* in different cancers on histological subtypes.

Cancers	Abbreviation	Comparison of type	Comparison	Statistical significance
Bladder urothelial carcinoma	BLCA	Individual cancer stages	Normal vs. Stage2	7.193200E-04
			Normal vs. Stage3	1.562000E-03
			Normal vs. Stage4	1.179660E-03
			Stage2 vs. Stage3	4.397100E-03
			Stage2 vs. Stage4	3.636500E-02
		Tumor histology	Normal vs. papillary tumors	4.441900E-04
			Normal vs. non-papillary tumors	9.622000E-04
			Papillary tumors vs. non-papillary tumors	1.318290E-03
Breast invasive carcinoma	BRCA	Individual cancer stages	Normal vs. Stage1	6.433300E-07
			Normal vs. Stage2	4.752000E-08
			Normal vs. Stage3	7.653900E-08
			Normal vs. Stage4	3.351400E-08
Kidney Chromophobe	KICH	Individual cancer stages	Normal vs. Stage1	6.939800E-03
			Normal vs. Stage2	7.504900E-03
			Normal vs. Stage3	2.763900E-03
			Normal vs. Stage4	2.385900E-02
Liver hepatocellular carcinoma	LIHC	Individual cancer stages	Normal vs. Stage1	1.351090E-04
			Normal vs. Stage2	1.303680E-03
			Normal-vs-Stage3	2.639000E-04
			Normal vs. Stage4	1.706019E-05
			Stage2 vs. Stage4	1.831740E-02
			Stage3 vs. Stage4	3.591600E-02
		Tumor histology	Normal vs. Hepatocellular carcinoma	2.412800E-04
Lung adenocarcinoma	LUAD	Individual cancer stages	Normal vs. Stage1	2.369099E-06
			Normal vs. Stage2	1.662889E-05
			Normal vs. Stage3	1.637189E-05
			Normal vs. Stage4	1.741339E-05
		Tumor histology	Normal vs. NOS	3.994800E-06
			Normal vs. vs. Mixed	1.047499E-06
			Normal vs. LBC-non-Mucinous	1.166550E-04
			Normal vs. SolidPatternPredominant	3.954100E-04
			Normal vs. Acinar	3.213199E-06
			Normal vs. LBC mucinous	3.558700E-05
			Normal vs. Mucinous carcinoma	2.459500E-03
			Normal vs. Papillary	1.842970E-04
			Normal vs. Mucinous	1.775199E-08
			Normal vs. Micropapillary	7.709399E-05
Mesothelioma	MESO	Individual cancer stages	Stage2 vs. Stage3	2.542300E-02
		Histological subtype	Biphasic-VS-Diffuse_malignant	1.356320E-02
			Diffuse_malignant-VS-Epithelioid	1.097310E-03
Testicular Germ Cell Tumors	TGCT	Tumor histology	Seminoma vs. Non Seminoma	1.160199E-10
Thymoma	THYM	Tumor histology	Type A vs. Type B1	2.937500E-03
			Type A vs. Type B3	2.885600E-02
			Type C vs. Type B1	3.228100E-02
			Type C vs. Type B3	3.883500E-02
			Type AB vs. Type B1	2.866100E-02
Thyroid carcinoma	THCA	Individual cancer stages	Normal vs. Stage1	1.482870E-03
			Normal vs. Stage2	3.703799E-05
			Normal vs. Stage3	3.422900E-03
			Normal vs. Stage4	3.224300E-03
			Stage1 vs. Stage2	1.996080E-02
		Histological subtype	Classical vs. Tall	3.723700E-02
			Classical vs. Normal	2.624000E-03
			Tall vs. Normal	1.219500E-04
			Follicular vs. Normal	5.565000E-04
			Other vs. Normal	3.395100E-04
Uterine Corpus Endometrial Carcinoma	UCES	Individual cancer stages	Normal vs. Stage1	5.819400E-03
			Normal vs. Stage2	2.637800E-02
			Normal vs. Stage3	3.095600E-02
			Normal vs. Stage4	2.793900E-02
		Histological subtype	Normal vs. Endometrioid	5.002900E-03
			Normal vs. Mixed serous and endometrioid	4.462500E-02
			Endometrioid vs. Serous	8.297800E-07

**Figure 5:**
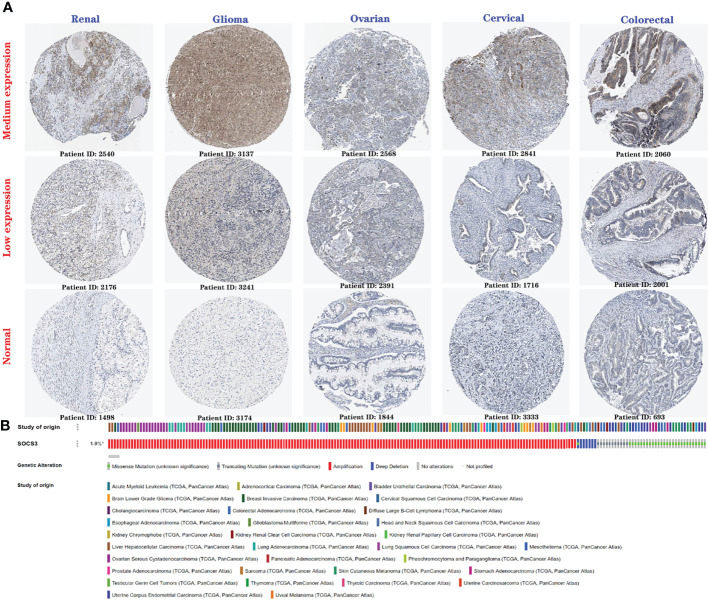
Protein expression level and genetic alteration of *SOCS3* in various cancers. **(A)** The protein expression of *SOCS3* in different cancers (low expression and medium expression) and normal tissues *via* Human Protein Atlas website. **(B)** Genetic variations of *SOCS3* in disparate cancers across OncoPrint. *altered/profiled=209/10950.

The protein expression levels of *SOCS3* in cancer tissues and the corresponding normal samples (medium expression and low expression) were explored *via* the Human Protein Atlas database. We found that the staining of *SOCS3* in the kidneys, ovaries, uterus, colon and rectum, and brain tumor specimens were higher than that in the normal tissues, which was consistent with the results of mRNA expression analysis, suggesting that high *SOCS3* expression may be a risk factor for LGG, GBM, COAD, KIRC, OV, and UCS (**Figure 5A**). Finally, we investigated the genomic alterations of SOCS3 in 10,967 patients across TCGA Pan-Cancer Atlas Studies using the cBioPortal database. The incidence of genetic variations in SOCS3 was increased by up to 1.9% (**Figure 5B**), and amplification was the most common type of *SOCS3* genetic alterations, followed by missense mutations, truncation mutations, and deep deletions. *SOCS3* amplification occurred in several cancers, among which the most common type of cancers was breast invasive carcinoma, followed by ovarian serous cystadenocarcinoma, liver hepatocellular carcinoma, lung squamous cell carcinoma, and LGG. In addition, gene mutation often occurred in the uterine corpus endometrial carcinoma, skin cutaneous melanoma, and colorectal adenocarcinoma. These findings indicated that SOCS3 is an oncogenic protein involved in cancer progression, different cancer stages, and various tissue subtypes, and can be employed as a potential biomarker for tumor diagnosis and treatment.

### Prognostic Values of *SOCS3* in Various Types of Cancer

**Figure 6:**
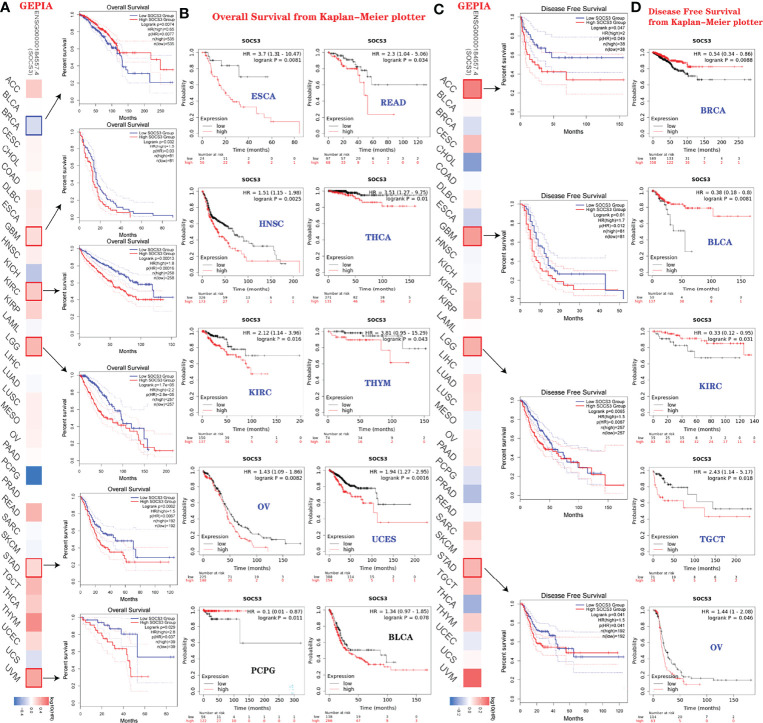
Relevance between *SOCS3* expression status and survival prognosis of various tumors in TCGA. **(A, C)** Analysis of the relationship between overall survival, disease-free survival, and *SOCS3* expression levels in different cancers *via* GEPIA2 website. **(B, D)** Same analysis using Kaplan–Meier database.

To further explore the potential prognosis of *SOCS3* in several types of cancer, GEPIA and Kaplan–Meier Plotter databases were used (32). Using GEPIA database, we observed that upregulation of *SOCS3* expression was relevant to poor overall survival rate in GBM (*p*=0.03), KIRC (*p*=0.00013), LGG (*p*=2.8e−5), STAD (*p*=0.0067), and UVM (*p*=0.037), whereas BRCA (p=0.0077) patients with high SOCS3 expression had higher overall survival rate (**Figure 6A**). DFS analysis displayed the relationship between upregulation of *SOCS3* expression levels and poor prognosis for TCGA cancer types, including ACC (*p*=0.049), GBM (*p*=0.012), LGG (*p*=0.0067), and STAD (*p*=0.041) (**Figure 6C**).

Moreover, the prognostic values of *SOCS3* in different types of cancer were also assessed using Kaplan–Meier Plotter databases. **Figures 6B** **, D** indicated that high *SOCS3* expression levels were associated with poor overall survival in ESCA (*p*=0.0081), READ (*p*=0.034), HNSC (*p*=0.0025), THCA (*p*=0.01), KIRC (*p*=0.016) THYM (*p*=0.043), OV (*p*=0.0082), and UCES (*p*=0.0016) (**Figure 6B**). In contrast, *SOCS3* expression status was relevant to a positive DFS in BRCA (*p*=0.0088), BLCA (*p*=0.0081), and KIRC (*p*=0.031), which were opposed to TGCT (*p*=0.018) and OV (*p*=0.046) (**Figure 6D**). In addition, the expression of *SOCS3* was relevant to AML, astrocytoma, glioma, breast cancer, colorectal cancer, ovarian cancer, and melanoma from the PrognoScan database (http://dna00.bio.kyutech.ac.jp/PrognoScan/index.html) (access date: November 15, 2021), which also indicated that *SOCS3* expression in BRCA and BLCA was associated with a positive prognosis (**Table 2**).

**Table 2:** Prognostic value of *SOCS3* in various cancers in PrognoScan database.

Dataset	Cancer type	Subtype	Endpoint	Cohort	N	Cox p-value	ln (HR)	HR [95% CI-low CI-up]
**GSE12417-GPL570**	Blood cancer	AML	Overall Survival	AMLCG (2004)	79	0.002015	-15.33	0.13[0.04-0.47]
**GSE4271-GPL96**	Brain cancer	Astrocytoma	Overall Survival	MDA	77	0.009723	1.17	1.80 [1.15 - 2.81]
**GSE4271-GPL97**	Brain cancer	Astrocytoma	Overall Survival	MDA	77	0.006450	1.08	1.27 [1.07 - 1.52]
**GSE4412-GPL97**	Brain cancer	Glioma	Overall Survival	UCLA (1996-2003)	74	0.022809	1.23	1.26 [1.03 - 1.54]
**GSE19615**	Breast cancer		Distant Metastasis Free Survival	DF/HCC	115	0.027466	-2.32	0.36 [0.14 - 0.89]
**GSE3143**	Breast cancer		Overall Survival	Duke	158	0.018826	0.94	1.60 [1.08 - 2.37]
**GSE2034**	Breast cancer		Distant Metastasis Free Survival	Rotterdam (1980-1995)	286	0.022263	-0.80	0.55 [0.33 - 0.92]
**GSE17536**	Colorectal cancer		Overall Survival	MCC	177	0.030321	-0.74	0.33 [0.12 - 0.90]
**GSE17537**	Colorectal cancer		Disease Specific Survival	VMC	49	0.032146	-1.70	0.10 [0.01 - 0.82]
**GSE8841**	Ovarian cancer		Overall Survival	Milan (1992-2003)	81	0.041825	0.84	1.81 [1.02 - 3.19]
**GSE17260**	Ovarian cancer		Overall Survival	Niigata (1997-2008)	110	0.024557	-0.89	0.56 [0.34 - 0.93]
**GSE19234**	Skin cancer	Melanoma	Overall Survival	NYU	38	0.013239	-1.73	0.30 [0.12 - 0.78]

In conclusion, we found that high *SOCS3* expression was associated with a poor prognosis in the majority of different types of cancer, and upregulated expression was associated with a poorer OS and DFS based on analyses using several databases.

### Association Between *SOCS3* Expression and Immune Infiltration in Cancer

**Figure 7:**
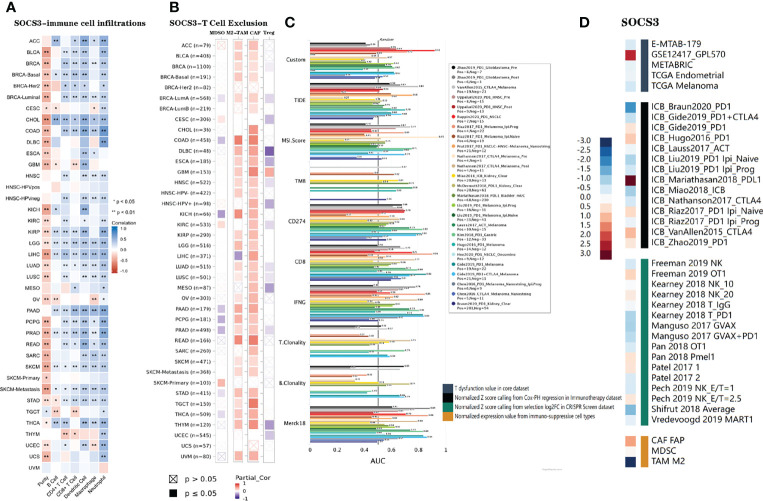
**(A, B)** Relevance between *SOCS3* expression status and six immune infiltration cells and four immunosuppressive cells in different cancers. Purity-corrected partial Spearman’s rho values and statistical difference were employed to account for the relationship. **(C)** Correlations between *SOCS3* and standardized tumor immune evasion markers in immune checkpoint blockade cohorts. The predictive abilities of the targets on the ICB response were assessed by AUC. **(D)** Correlations between *SOCS3* and lymphocyte-mediated tumor killing in T dysfunction, immunotherapy, CRISPR screen, and immune-suppressive cell types. MDSCs, myeloid-derived suppressor cells; M2-TAMs, M2 subtype of tumor-associated macrophages; CAFs, cancer-associated fibroblasts; Tregs, regulatory T cell. **p < 0.01, *p < 0.05.

We assessed tumor immune infiltration from 39 TCGA cancers, and seven of these exhibited a notable positive relationship between *SOCS3* expression levels and infiltration of six kinds of immune cells (B cells, CD8 + T cells, CD4 + T cells, DCs macrophages, and neutrophils), including CHOL, LGG, LIHC PAAD, PRAD, SKCM, and STAD. Tumor immunity infiltration of LGG, LIHC, PAAD, and PRAD were strongly correlated with *SOCS3* expression (*p*<0.01), while GHOL, SKCM, SKC-metastasis, and STAD were relatively weakly correlated with infiltration of the six types of immune cells (*p*<0.05). No significant correlation (*p*>0.05) or negative correlation (*r*<0, *p*<0.05) was observed between all the other types of cancer and tumor immune infiltration of the six kinds of immune cells (**Figure 7A**).

Four types of immunosuppressive cells promoting T-cell exclusion, including MDSCs, CAFs, M2-TAMS, and Treg cells, were used to predict associations with *SOCS3* expression. We discovered that the expression of *SOCS3* was positively relevant to tumor infiltration of MSDCs in CESC, KIRC, and SKCM-Primary, whereas it was negatively correlated with BRCA-LumA, COAD, KICH PAAD, PCPG, PRAD, STAD, and THCA. Tumor infiltration of M2-TAMs occurred in ACC, BLCA, BRCA, COAD, DLBC, ESCA, GBM, HNSC, KICH, KIRP, LGG, LUAD, LUSC, OV, PAAD, PCPG, PRAD, PEAD, SKCM, STAD, TGCT, THYM, and UVM; tumor infiltration of Tregs occurred in DLBC, GBM, CESC, ESCA, HNSC, MESO, PRAD, THYM, and UCEC; and tumor infiltration of CAFs occurred in almost all of the 40 TCGA types and subtypes, except CESC, UCEC, BRCA-Her2, UCS, and SKCM-Primary (**Figure 7B**).

We predicted the biomarker association between *SOCS3* with standardized biomarkers according to the response results and OS of the ICB groups. We observed that *SOCS3* contained an area under the receiver operating characteristic curve (AUC) of >0.5 in 12 of the 25 ICB groups. The predicted value of *SOCS3* was higher than that of TMB, T.Clonality, and B.Clonality that had AUC values of >0.5 in eight, nine, and seven ICB sub-cohorts. However, *SOCS3* had a lower predictive value than TIDE, MIS.Score, CD274, CD8, IFNG, and Merck 18 (**Figure 7C**).

The TIDE database also indicated that high *SOCS3* expression was related to unfavorable programmed death 1 protein (PD1) therapeutic consequences in melanoma (ICB_Gide2019_PD1), melanoma (ICB_Hugo2016_PD1), melanoma (ICB_Riza2017_PD1) and glioblastoma (ICB_Zhao2019_PD1), PD-ligand 1 (L1) therapeutic consequences in bladder urothelial carcinoma (ICB_Mariathasan2018_PD-L1), and CTLA4 in melanoma (ICB_VanAllen2015_CTLA4), whereas it was associated with positive therapeutic outcomes in kidney renal clear cell carcinoma (ICB_Braun2020_PD1), kidney renal clear cell carcinoma (ICB_Braun2020_PD1), melanoma (ICB_Nathanson2017_CTLA4), and melanoma (ICB_Lauss2017_ACT). Gene knockout phenotype analysis of CRISPR screening indicated that *SOCS3*-knockout was a significant influencing factor of lymphocyte-mediated tumor killing in B16 melanoma (Freeman_2019_NK) and MC38 colon cancer (Kearney2018_T_PD1) models. Furthermore, upregulation of *SOCS3* expression levels was relevant to T dysfunction in leukemia (GSE12417_GPL570) (**Figure 7D**).

### Epigenetic Modifications of the *SOCS3* Gene are Related to Dysfunctional T-Cell Phenotypes and Poor Outcomes in Several Types of Cancer

**Figure 8:**
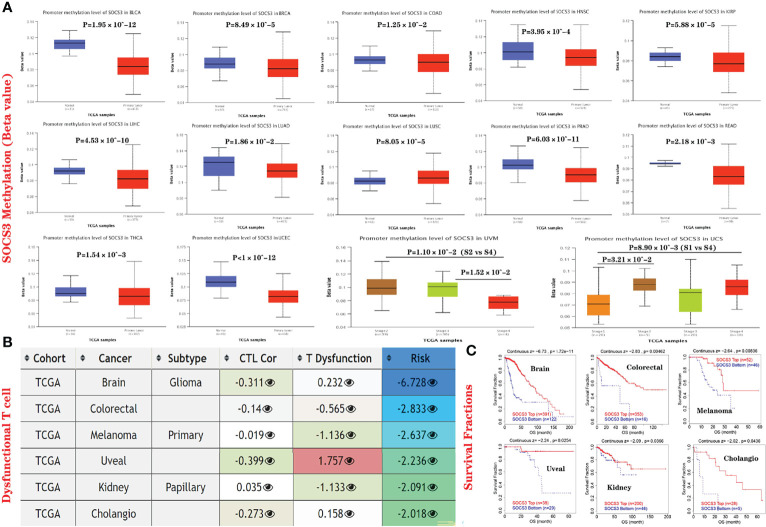
Epigenetic modifications of *SOCS3* are related to dysfunctional T-cell phenotypes and poor outcomes in various cancers. **(A)** UALCAN database was employed to investigate the *SOCS3* methylation levels (beta values) in various tumors *via* TCGA dataset, such as BLCA, BRCA, COAD, HNSC, KIRP, LIHC, LUAD, LUSC, PRAD, READ, THCA, UCEC, UVM, and UCS. **(B)** Correlations between *SOCS3* methylation and cytotoxic T-cell levels (CTLs), dysfunctional T-cell phenotypes, and risk factors in TCGA cancers. **(C)** Effect of *SOCS3* methylation levels on overall survival in TCGA cancer cohorts. We only indicated statistically significant cancers.

The UALCAN website was used to explore the DNA methylation levels of *SOCS3* in various cancers. In various types of cancer, it reminded hypomethylated, such as in BLCA, BRCA, COAD, HNSC, KIRP, LIHC, LUAD, LUSC, PRAD, READ, THCA, and UCEC (**Figure 8A**). These results strongly suggested that SOCS3 methylation is negatively associated with its mRNA expression levels in different types of tumors. Studies have shown that after methylation of CpG islands in the SOCS3 promoter region, SOCS3 gene silencing, and mRNA expression decreased, which was predictive of adverse clinical outcomes and prognosis, such as GBM (33–35). To further investigate the influence of promoter methylation level of S*OCS3* in different types of cancer, the TIDE website was used, and it was shown that hypermethylation of *SOCS3* was negatively related to dysfunctional T-cell phenotypes (**Figure 8B**) and longer survival durations of patients with colorectal, melanoma, uveal, and kidney cancers (**Figure 8C**) Thus, the methylation status of *SOCS3* in several types of cancer may be related to dysfunctional T-cell phenotypes through several mechanisms and may be predictive of a less favorable prognosis in colorectal carcinoma, melanoma, uveal melanoma, and kidney cancer.

### Analysis of the Genetic Alterations and Carcinogenic Characteristics of *SOCS3*

**Figure 9:**
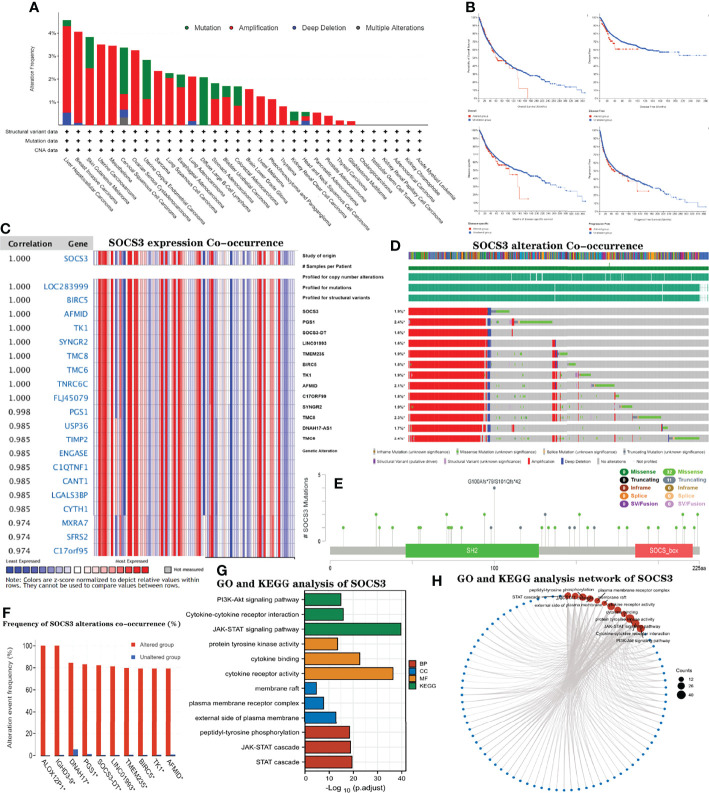
Genetic diversities and oncogenic characteristics of *SOCS3* co-occurrence with dysregulation of its functional chaperone and may predict poor prognosis of cancers. The mutation features of *SOCS3* in TCGA cancers were explored *via* cBioPortal database, **(A)** displayed the mutation frequency and **(E)** showed the mutation site. **(B)** Kaplan–Meier curves of the influence of *SOCS3* alterations on OS, DSS and PFS. **(C)** Heatmap indicated *SOCS3* overexpression co-occurrence *via* Oncomine database. Spearman’s rho value and estimated statistical significance were employed to reflect the degree of relevance between these genes. **(D)** Displayed *SOCS3* alteration co-occurrence genes, including *PGS1, SOCS3-DT, LINC01993, TMEM235, BIRC5, TK1, AFMID, C17ORF99, SYNGR2, TMC8, DNAH17-AS1*, and *TMC6.* **(F)** Frequencies of *ALOX12P1, IGHD3-9, DNAH17, PGS1, SOCS3-DT, LINC01993, TMEM235, BIRC5, TK1*, and *AFMID* alteration co-occurrence with *SOCS3* alterations. **(G, H)** Co-occurrence GO and KEGG enrichment analysis of *SOCS3* in different cancers.

We used the cBioPortal database to compare the types and frequencies of genetic changes in the *SOCS3* gene in the different types of cancer. *SOCS3* gene amplification and mutations were the main types of alterations observed. However, deep deletions and duplication alterations were rare (**Figure 9A**). **Figure 9E** shows the sites, types, and case numbers of *SOCS3* genetic mutations, and we observed that missense mutation of *SOCS3* was the most common type of alteration, followed by truncation mutations. These changes could have significant influences on a patients’ OS, DFS, and progression-free survival (**Figure 9B**). The Oncomine website was employed to analyze the genes associated with *SOCS3* in the cancer cohorts. *SOCS3* expression was highly correlated with several genes, including *TK1, BIRCS, AFMID, SYNGR2, TMC8*, and *TMC6* (*r*≥0.99) (**Figure 9C**). As for these *SOCS3*-related genes, we found that they were associated with tumor progression and their expression was increased during tumorigenesis. For example, the *mRNA* and protein levels of *TK1* in liver tissues and the serum of patients with liver cancer were significantly increased, while the *TK1* target gene knockdown had significant anti-apoptotic and proliferative effects on HepG2 cells (36). Interestingly, these gene alterations were similar to those of *SOCS3* (**Figures 9D**–**F**), suggesting that these genes are functional partners associated with carcinogenic effects of *SOCS3* in tumors. Gene enrichment analysis was employed to explore the biological processes modulated by these genes (**Figures 9G** **, H**). GO pathway showed that this group of genes primarily regulated the *STAT* cascade, *JAK/STAT* cascade, and peptidyl-tyrosine phosphorylation, and KEGG enrichment analysis included cytokine-cytokine receptor interaction, *JAK/STAT*, and *PI3K/Akt* signaling pathways (**Table 3**). Together, the carcinogenic effects of *SOCS3* were related to the regulation of *PGS1, BIRC5, TK1, AFMID*, and, other genes associated with the development of cancer.

**Table 3:** GO and KEGG enrichment analysis of *SOCS3* and their interactors.

Ontology	ID	Description	GeneRatio	BgRatio	p-value	p.adjust	q-value
BP	GO:0097696	STAT cascade	22/113	166/18670	1.33e-23	3.98e-20	2.43e-20
BP	GO:0007259	JAK-STAT cascade	21/113	156/18670	1.05e-22	1.58e-19	9.64e-20
BP	GO:0018108	peptidyl-tyrosine phosphorylation	27/113	363/18670	4.42e-22	4.11e-19	2.52e-19
CC	GO:0009897	external side of plasma membrane	22/113	393/19717	6.07e-16	1.28e-13	1.12e-13
CC	GO:0098802	plasma membrane receptor complex	15/113	295/19717	1.44e-10	1.51e-08	1.33e-08
CC	GO:0045121	membrane raft	12/113	315/19717	2.63e-07	1.43e-05	1.26e-05
MF	GO:0004896	cytokine receptor activity	28/114	96/17697	1.17e-39	3.30e-37	2.46e-37
MF	GO:0019955	cytokine binding	22/114	128/17697	1.28e-25	1.81e-23	1.35e-23
MF	GO:0004713	protein tyrosine kinase activity	16/114	134/17697	3.22e-16	2.56e-14	1.91e-14
KEGG	hsa04630	JAK-STAT signaling pathway	40/102	162/8076	1.05e-42	1.91e-40	7.60e-41
KEGG	hsa04060	Cytokine-cytokine receptor interaction	29/102	295/8076	1.18e-18	1.08e-16	4.30e-17
KEGG	hsa04151	PI3K-Akt signaling pathway	30/102	354/8076	1.80e-17	1.10e-15	4.35e-16

CC, cellular component; GO, gene ontology; KEGG, Kyoto Encyclopedia of Genes and Genomes; MF, molecular functions. p. adjust method = “FDR.” BgRatio: the number of genes of the corresponding species contained in the corresponding GO database/the number of genes of the corresponding species contained in GO database.

### Further Enrichment Analysis of *SOCS3*-Related Genes

**Figure 10:**
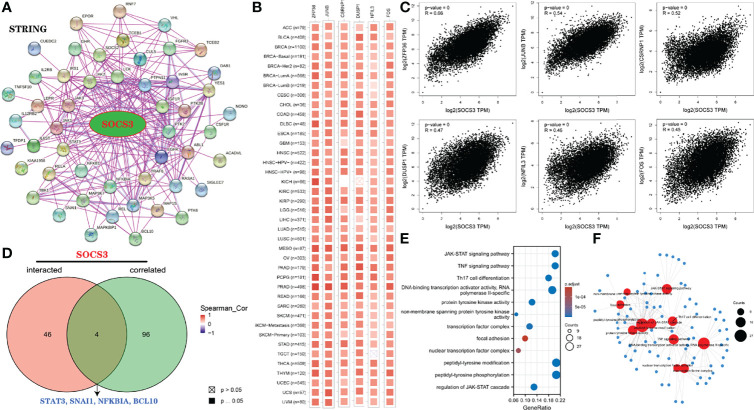
Enrichment analysis of *SOCS3*-related genes. **(A)** *SOCS3*-binding proteins *via* STRING dataset. **(B, C)** One hundred genes related to *SOCS3* were obtained *via* GEPIA2 database, and 6 genes most related to *SOCS3* were specifically analyzed, including *ZFP36, JUNB, CSRNP1, DUSP1, NFIL3*, and *FOS*. Then, the heatmap corresponding to these genes were analyzed in detail. **(D)** Intersection genes of *SOCS3*-binding and correlated genes. **(E, F)** GO and KEGG analysis based on *SOCS3*-binding genes and interacted genes.

Next, to explore the molecular mechanism in which *SOCS3* participated in to promote the tumorigenesis of various tumors, we used the STRING dataset to construct a PPI network for *SOCS3* (**Figure 10A**). The interaction network contained 50 *SOCS3*-binding proteins. Next, the GEPIA2 was used to acquire the top 100 genes associated with *SOCS3* expression across the types of cancer assessed. Specifically, we selected six genes with the highest correlation with *SOCS3* expression, including *ZFP36* ring finger protein (*ZFP36*) (*r*=0.66), *JunB* proto-oncogene, *AP-1* transcription factor subunit *(JUNB*) (*r*=0.54), cysteine and serine-rich nuclear protein 1 (CSRNP1) (*r*=0.52), dual specificity phosphatase 1 (DUSP1) (*r*=0.47), nuclear factor, interleukin 3 regulated (*NFIL3*) (*r*=0.46) and *Fos* proto-oncogene, *AP-1* transcription factor subunit (*FOS*) (*r*=0.45) genes (all *p*<0.001), and we found that these genes were positively associated with *SOCS3* (**Figures 10B** **, C**). Additionally, as can be seen from **Figure 10B**, these genes were also positively related to majority of cancer types. Finally, we took the intersection of the two groups of genes and found four common genes: the snail family transcriptional repressor 1 (*SNAI1*), signal transducer and activator of transcription 3 (*STAT3*), *NFKB* inhibitor α (*NFKBIA*), and *BCL10* immune signaling adaptor (*BCL10*) (**Figure 10D**).

Furthermore, GO and KEGG analysis were performed using these two groups to explore the effect of *SOCS3* on cancer pathogenesis. As shown in **Figures 10E, F**, *SOCS3* and their co-operators were significantly related to the *JAK/STAT* and *TNF* signaling pathways, Th17 cell differentiation, *DNA*-binding transcription activator activity, *RNA* polymerase II-specific, peptidyl-tyrosine phosphorylation, regulation of *JAK/STAT* cascade, protein tyrosine kinase activity, transcription factor complex, and peptidyl-tyrosine modification, among others.

### *SOCS3* Expression Is Relevant to the Treatment of Various types of Cancer

**Figure 11:**
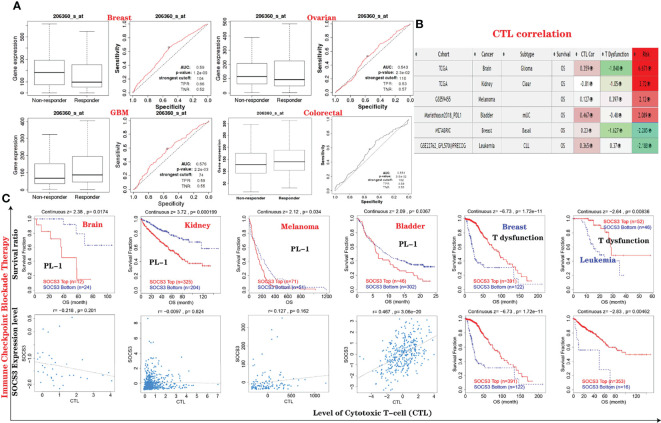
*SOCS3* expression is relevant to the treatment of disparate cancers. **(A)** Relationship between *SOCS3* expression and responses to chemotherapy in breast, ovarian, brain and colorectal cancers, which is displayed *via* ROC curve plot. **(B, C)** Employed TIDE database to indicate the correlations between immunotherapeutic response (ICB) and cytotoxic T-cell level (CTL) and *SOCS3* expression levels in TCGA cancers using Kaplan–Meier curves. Only cancers with statistically significant differences were listed.

We used the ROC plotter website to investigate the effects of *SOCS3* expression on chemotherapy response in various types of cancer. We found that breast and ovarian cancer patients with high expression levels of *SOCS3* were resistant to chemotherapy; however, GBM and colorectal cancer patients with high expression levels of *SOCS3* were more sensitive to chemotherapy than patients with low *SOCS3* expression (**Figure 11A**). Furthermore, using the TIDE website, it was determined that patients with downregulated expression levels of *SOCS3* had a higher overall survival rate after ICB (*PD-1* or *PD-L1*) treatment among kidney cancer, melanoma, and bladder cancer patients. It is worth mentioning that *SOCS3* expression levels were negatively relevant to CTL in glioblastoma patients, which indicated an interaction with T-cell exclusion. For breast cancer and leukemia patients with T-cell dysfunction, the cohort with high *SOCS3* expression possessed a higher survival time (**Figures 11B** **, C**).

### Experimental Verification of *SOCS3* Expression in GBM and LGG by IHC

**Figure 12:**
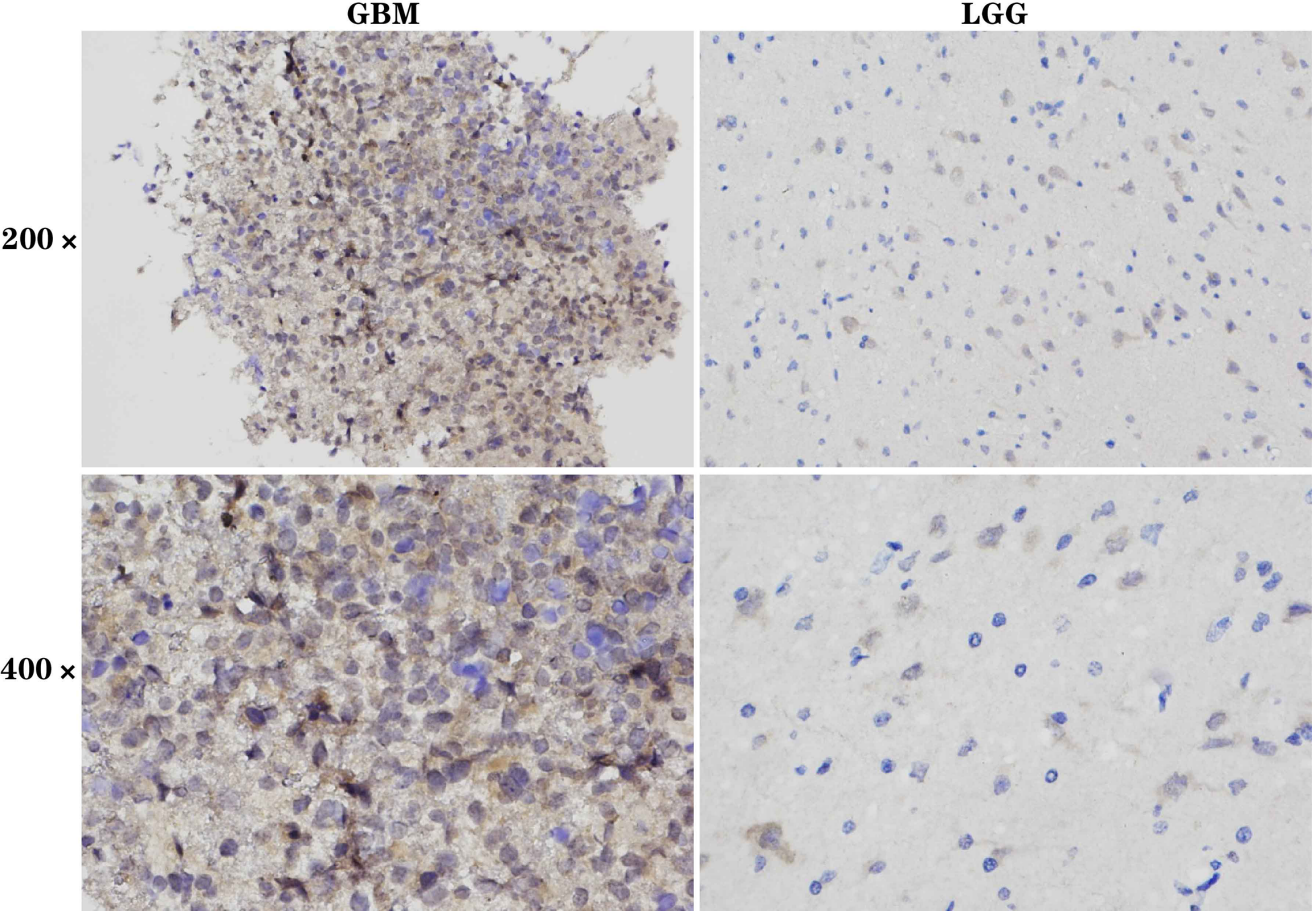
The experimental validation of *SOCS3* by IHC. Expression of *SOCS3* in GBM and LGG tissues is shown at 200× and 400× magnification, respectively. It is found that *SOCS3* is highly expressed in GBM and low expressed in LGG.

Immunohistochemistry (IHC) was used to assess 30 HGG patients’ tissues (11 GBM) and 32 LGG patients’ samples (**Figure 12**). We observed *SOCS3* expression in tissue specimens using low-power field (200×) and high-power field (400×), respectively, and the results indicated that *SOCS3* expression was significantly increased in the HGG tissues compared to the LGG samples, consistent with the results of the bioinformatics analysis (**Figure 12**) (**Supplementary Table S1**).

## Discussion

The results of this study indicated that *SOCS3* was strongly associated with the occurrence and development of different types of cancer. Several studies have also found that *SOCS3* is closely related to various types of cancer (10, 37–39). Whether *SOCS3* exerts a role in the pathogenesis and therapeutic response of the different types of cancer *via* common molecular pathways remains to be determined. In this study, we revealed the relationship between *SOCS3* expression and genetic alterations with the survival status, tumor microenvironment, tumor cell and immune cell invasion, the related molecular pathways, the therapeutic responses, and the prognosis in 39 different types of cancer based on data obtained from TCGA.

Phenotypic and functional heterogeneity can occur between different types of tumors and between cancer cells within the same tumor due to alterations in the genetics, the environment, and the cellular characteristics (40). The differences in protein expression may provide a reference for the diagnosis, therapy, and prognosis of tumors (41). We explored the carcinogenic role of *SOCS3* in several types of cancer through *mRNA* and protein expression level analysis and found that *SOCS3* was upregulated or downregulated in almost all tumor types, and in a variety of tumors, its expression was related with the tumor stage and/or histological subtype, such as BLCA, THCA, and MESO. These results indicate that *SOCS3* is relevant to the occurrence and development of several types of cancer and maybe an underlying target for the diagnosis and therapy of different kinds of cancers. However, survival prognostic analysis showed that abnormal *SOCS3* expression was varied among the different tumors. For example, GBM patients with high expression of *SOCS3* has a poorer OS, while KIRC patients exhibited the opposite result, and these results were consistent with previous *in vivo* and *in vitro* studies (13, 42).

The results of the present study showed that the difference in *SOCS3* expression was not statistically significant in several types of tumors, including CESC, CHOL, COAD, DLBC, KICH, KIRP, LAML, LIHC, LUAD, LUSC, MESO, PAAD, PRAD, PEAD, SARC, SKCM, UCS, and UVM, which suggests that *SOCS3* expression was not relevant to the signaling imbalance in these tumors. However, genetic alterations in *SOCS3* may disrupt the immune micro-environment and affect the prognosis of patients.

Tumor immune microenvironment and immune evasion are relevant to therapeutic and prognostic responses in cancer (43). Tumor invasion of immune cells can promote tumor development and result in a dysfunctional T-cell phenotype (44, 45). Our research showed that the expression of *SOCS3* was relevant to immune infiltration in seven kinds of cancer, including CHOL, LGG, LIHC, PAAD, PRAD, SKCM, and STAD. In addition, Tregs, MDSCs, CAFs, and M2-TAMS are the main mechanisms of T-cell exclusion (46). Our research found that *SOCS3* expression is relevant to tumor-promoting immune cells of almost all types of cancer; therefore, T-cell exclusion may be a primary mechanism of tumor immune cell infiltration. Our research also found that *SOCS3* methylation levels were negatively associated with mRNA expression in various tumors.

We explored alterations in frequency and types of *SOCS3* in different types of cancer and found that *SOCS3* gene amplification and mutation were the most common alterations, among which missense mutation was the most common mutation type. These results suggest that cancer progression may be driven by genetic changes and the accumulation of alterations in gene expression patterns (47). Alterations in *SOCS3* gene expression profiles may be associated with a poorer prognosis in the majority of cancers. This oncogenic effect cannot be attributed to a single genetic change but may instead be the result of a combination of multiple related genes. We found that several genes were highly correlated with *SOCS3* expression and may play similar pathogenic roles, such as *TK1, BIRSC, TMC8*, and *AFMID* (48). These consequences reveal that the carcinogenic influence of *SOCS3* may be caused by the interaction of multiple functional partners. Furthermore, we performed enrichment analysis of *SOCS3* and its functional partners to explore the possible pathogenic mechanism of *SOCS3* and found that the *JAK/STAT* and other signaling pathways were involved in the SOCS3-mediated tumor progression, consistent with existing research results (37, 49, 50). However, some biological functions require further experiments to elucidate the relevance of such as the correlation between SOCS3 and peptidyl-tyrosine phosphorylation.

Our research indicated that ovarian and breast cancer patients with high *SOCS3* expression levels were resistant to chemotherapy; however, GBM and colorectal cancer patients were more sensitive to chemotherapy. Anti-*PD-1* or *PD-L1* antibodies can efficaciously remedy a variety of cancers (51), and the results of the present study showed that certain patients with low *SOCS3* expression achieved better prognoses after ICB (*PD-1* or *PD-L1*) treatment, such as in patients with kidney cancer, melanoma, and bladder cancer. The *SOCS3* expression level was negatively correlated with CTL in some tumor patients, suggesting interactions with T-cell exclusion. Together, the results show that *SOCS3* is an immuno-oncogenic molecule that may serve as a marker for tumor diagnosis, treatment, and prognosis, and thus, further experimental validation to ascertain its value is justified.

## Conclusion

In conclusion, our research indicates that *SOCS3* was valuable for diagnosis, staging, histological subtyping, prognosis, and therapeutic response of several types of cancer in the context of immuno-oncology through different mechanisms, and those genetic or epigenetic alterations in the *SOCS3* genes were often predictive of a poor prognosis. Alterations in *SOCS3* expression and tumor cell immune infiltration were also predicted by the presence of immunosuppressive cells (MDSCs, CAFs, M2-TAMS, and Treg), which promote T-cell exclusion. Furthermore, we found that the methylation status of *SOCS3* was relevant to dysfunctional T-cell phenotypes and a poor prognosis in cancer. The carcinogenic characteristics of *SOCS3* are also closely related to multiple functional partner genes and various biological functions. *SOCS3* expression was also shown to have certain effects on the response to chemotherapy and immunotherapy in several types of cancer, and additional experiments are required to further elucidate the mechanisms involved. In summary, *SOCS3* may be a potential marker for diagnosis, therapy, prognosis, and follow-up in several types of cancer.

## Data Availability Statement

The datasets presented in this study can be found in online repositories. The names of the repository/repositories and accession number(s) can be found in the article/**Supplementary Material**.

## Ethics Statement

The studies involving human participants were reviewed and approved by Ethics Review Committee of the Fifth Affiliated Hospital of Zhengzhou University. The patients/participants provided their written informed consent to participate in this study.

## Author Contributions

LD wrote the manuscript. YT, ZS, WL, WH, SZ, ZX, and XG revised it critically for important intellectual content and gave important advice. XW provided the overall idea of the article and revised the original manuscript. All authors read and approved the manuscript and agree to be accountable for all aspects of the research in ensuring that the accuracy or integrity of any part of the work are appropriately investigated and resolved.

## Funding

The present study was supported by grants from the National Natural Science Foundation of China (grant nos. 81972361 and 81874068) and Medical Science and Technology Project of Henan Province (grant nos. LHGJ20210487 and 222102310039).

## Conflict of Interest

The authors declare that the research was conducted in the absence of any commercial or financial relationships that could be construed as a potential conflict of interest.

The handling editor YZ declared a shared parent affiliation with the authors at the time of review.

## Publisher’s Note

All claims expressed in this article are solely those of the authors and do not necessarily represent those of their affiliated organizations, or those of the publisher, the editors and the reviewers. Any product that may be evaluated in this article, or claim that may be made by its manufacturer, is not guaranteed or endorsed by the publisher.
