# Supplementary material for: SOCS3 Acts as an Onco-immunological Biomarker With Value in Assessing the Tumor Microenvironment, Pathological Staging, Histological Subtypes, Therapeutic Effect, and Prognoses of Several Types of Cancer
Source: Front Oncol. 2022 May 6;12:881801. doi: 10.3389/fonc.2022.881801 (PMC9122507; doi:10.3389/fonc.2022.881801)
Supplement: Supplementary file 1 [file Table_1.docx]

**SUPPLEMENTARY TABLE S1 |** Tumor sample characteristics

| Histopathological parameter | | Samples, n=62 |
| --- | --- | --- |
| Grade (LGG: 1-2; HGG: 3-4) | 1 | 6 |
|  | 2 | 26 |
|  | 3 | 19 |
|  | 4 (GBM) | 11 |
| Age | ＜40 years | 17 |
|  | ≥40 years | 45 |
| SOCS3 IHC score | HGG (20) (GBM:11) | 2+, n=6 |
|  |  | 3+, n=19 |
|  | LGG (32) | 0, n=7 |
|  |  | 1+, n=27 |
|  |  | 2+, n=3 |

Immunohistochemistry staining intensities showed weak, moderate to strong cytoplasmic positivity in SOCS3 IHC scores of 1+, 2+ and 3+ glioma cases.
